# Copper oxide nanoparticles doped with lanthanum, magnesium and manganese: optical and structural characterization

**DOI:** 10.1098/rsos.220485

**Published:** 2022-11-16

**Authors:** Maribel Guzman, Wei Tian, Chantal Walker, Jose E. Herrera

**Affiliations:** ^1^ Department of Engineering, Pontifical Catholic University of Peru, Avenida Universitaria 1801, Lima 15088, Peru; ^2^ Department of Chemical and Biochemical Engineering, Western University, London, ON, N6A 5B9, Canada

**Keywords:** cuprous oxide nanoparticles, band gap, lanthanum, magnesium, manganese

## Abstract

Copper oxide (Cu_2_O) is a promising semiconductor for photovoltaic and photocatalytic applications since this material has a high optical absorption coefficient and lower band gap (2.17 eV). Doped lanthanum (La), magnesium (Mg) and manganese (Mn) Cu_2_O nanoparticles (Cu_2_O Nps) were prepared by a displacement reaction. The doped and undoped Cu_2_O Nps were characterized with scanning electron microscopy–energy dispersive X-ray spectroscopy (EDS), X-ray diffraction (XRD), transmission electron microscopy (TEM) and ultraviolet–visible spectroscopy. The EDS results confirm the presence of La, Mg and Mn in the Cu_2_O Nps. The XRD results confirm the formation a single cubic phase of Cu_2_O with a cuprite structure. TEM images confirm the formation of Nps with mean diameters between 12.0 ± 6.1 and 30.8 ± 11.0 nm. Doped and undoped Nps present a narrow band gap (2.40 eV), blue shifted with respect to bulk Cu_2_O.

## 1. Introduction

In the last two decades, the study of new oxide-based semiconductors has greatly intensified, since these materials can be used in solar cells and transparent electronics [1]. In fact, nanoparticles (Nps) of diverse metal oxides have shown potential applications in optical devices, purification systems, biomedical systems, photocatalysis and photovoltaics. Among promising metal oxide formulations such as tin oxide (SnO_2_) [2–5], titanium dioxide (TiO_2_) [6–8], tungsten oxide (WO_3_) [9–11], vanadium pentoxide (V_2_O_5_) [12–14] and zinc oxide (ZnO) [15–19], cuprous oxide (Cu_2_O) has emerged as an interesting alternative to study due to its optical properties. Indeed, Cu_2_O is an important p-type metal oxide semiconductor with a band gap of 2.17 eV, which makes it a promising material for the conversion of solar energy into electricity [20,21]. Cu_2_O exhibits a cubic structure and a p-type conductivity arising from copper vacancies that introduces an acceptor level of approximately 0.5 eV above the valence band [22]. Therefore, Cu_2_O is considered a suitable material for photovoltaic applications [1]. In addition to its special optical and electrical properties, Cu_2_O has other advantages such as non-toxicity and, compared to other oxides (Fe_2_O_3_, In_2_O_3_, NiFe_2_O_4_, NiO, Sb_2_O_3_, SnO_2_, ZnO, V_2_O_5_ and others), its low cost [23–28].

In semiconductors and insulators, electrons are confined to a number of energy bands and are forbidden in other regions. The term ‘band gap’ refers to the difference in energy between the top of the valence band and the bottom of the conduction band [29]. Since for semiconductors the band gap can be small, doping with other elements, even in very small amounts, can modify this gap and drastically increase the conductivity of the material. In particular, it is possible to adjust the bandgap by modifying or partially substituting a transition metal with another element. Indeed, doping of p-type (NiO) or n-type (ZnO) semiconductor nanostructures has been previously reported by Das *et al*. [30], Bhatt *et al*. [31], Manikandan *et al*. [32,33] and Shah *et al*. [15]. However, the exciting physical properties of transition metal oxides arising from the d-electrons can also be affected or disappear with the modification of this band gap [34]. For this reason, researchers have tried to improve the electrical and optical properties of Cu_2_O by incorporating various transition metals such as cobalt [35], iron [35,36], manganese (Mn) [22,35,37–40] and nickel [35]. In addition, the incorporation of other elements such as aluminium [41], cerium [42], lithium [43–45], magnesium (Mg) [1,43,46–48], silver [41], sodium [44], samarium [49], titanium [44] and zinc [42,43,50] has also been evaluated.

There are many methods to obtain doped Cu_2_O nanostructures, such as biosynthesis [51,52], chemical vapour deposition [46,47], electrodeposition [22], hydrothermal synthesis [37], pulsed laser deposition [40], solvothermal synthesis [38,39] and spray pyrolysis [46,48]. In addition to these methods, the chemical displacement or substitution technique is an interesting but little-explored alternative. This technique involves a reaction in which one element is replaced by another within a chemical compound [53].

The present work reports the preliminary results of the synthesis of Cu_2_O Nps doped with lanthanum (La), Mg and Mn through a chemical shift reaction, and the characterization of the resulting materials including the evaluation of their band gap energy.

## 2. Experimental section

### 2.1. Materials and methods

#### 2.1.1. Chemicals

Sodium hydroxide, NaOH (97%), was purchased from Alfa Aesar (Toronto, Canada). La(NO_3_)_3_.6H_2_O (99%), Mg, Mg(NO_3_)_2_.6H_2_O (99%), and Mn, Mn(NO_3_)_2_.nH_2_O (98%), were purchased from Sigma-Aldrich (Toronto, Canada). Absolute ethanol, EtOH (99%), was also obtained from Sigma-Aldrich (Toronto, Canada). All chemicals were used as received without further purification. In all the experimental syntheses, Milli-Q water (18 MΩ cm) obtained from a purification system (Millipore, Darmstadt, Germany) was used.

#### 2.1.2. Preparation of doped copper oxide nanoparticles

The modified chemical reduction method proposed by Badawy *et al.* [54] was used for the synthesis of Cu_2_O Nps [55,56]. For the synthesis, ethanolic solutions of sodium hydroxide (20 mM), Mg nitrate (20 mM), Mn nitrate (20 mM) and La nitrate (20 mM) were used. Approximately 100 mg of previously synthesized Cu_2_O Nps was suspended in 30 ml of ethanol. The solution containing the dopant element was gradually added to the suspension of Nps under magnetic stirring. The process was carried out for 120 min at 60°C. At the end of the reaction, the colloids were centrifuged at 3000 rpm for 5 min using a microcentrifuge (Thermo Scientific, ST-40R). The Nps were placed in a desiccator. To characterize the samples obtained, the Nps were suspended in absolute ethanol using an ultrasonic cleaning container (Fisher Bioblock Scientific).

### 2.2. Characterization techniques

#### 2.2.1. Structural characterization

To perform the structural analysis, a Bruker D5000 theta-theta X-ray diffractometer (Karlsruhe, Germany) equipped with a copper anticathode (*λ* Cu K*α* = 1.5418 Å) was used. Data were collected over the range 2*θ* = 25°–80° using a step size of 0.02° and a count time of 1 s per step. The X-ray diffraction (XRD) reference intensity ratio methodology was used to assign the phases observed in the diffractograms.

#### 2.2.2. Elemental composition analysis

Chemical composition was determined by scanning electron microscopy (SEM) using an FEI Quanta 650 in secondary electron mode at an accelerating voltage of 3–10 kV (FEI Europe BV; Eindhoven, The Netherlands). Energy-dispersive X-ray spectroscopy (EDS) analysis was performed with an Ametek EDAX TEAM system coupled to an SEM microscope.

#### 2.2.3. Morphological analysis

The size and morphology of the doped and undoped Cu_2_O Nps were analysed by transmission electron microscopy (TEM). TEM analysis was carried out using a Philips CM10 TEM instrument, upgraded with a digital AMT system and a LaB6 filament. The acceleration voltage used was 80 kV. Before analysis, dried samples were suspended in 2-propanol, and a drop of the suspension was spotted on a copper TEM grid coated with lacey carbon film.

#### 2.2.4. Surface area and pore size distribution

The specific surface area was determined using Brunauer–Emmett–Teller (BET) measurements. Nitrogen adsorption–desorption was developed at 77.35 K in an ASAP 2010 Analyzer. Before each measurement, 150–250 mg of sample was degassed at 200°C for 2 h and until the pressure was below 5 mmHg. Adsorption isotherms were measured within a relative pressure range of 0.001 to 1 at 77.35 K.

#### 2.2.5. Optical properties

Ultraviolet–visible–near-infrared (UV-VIS NIR) spectra were recorded in diffuse reflectance mode using a Shimadzu UV-VIS NIR spectrometer equipped with a Praying Mantis diffuse reflectance cell (Harrick Scientific). The spectra were referenced to a Spectralon standard (DRP-SPR, Harrick Scientific).

## 3. Results and discussion

The Cu_2_O Nps doped using a modified displacement reaction were characterized by the techniques described previously.

**Figure 1.**
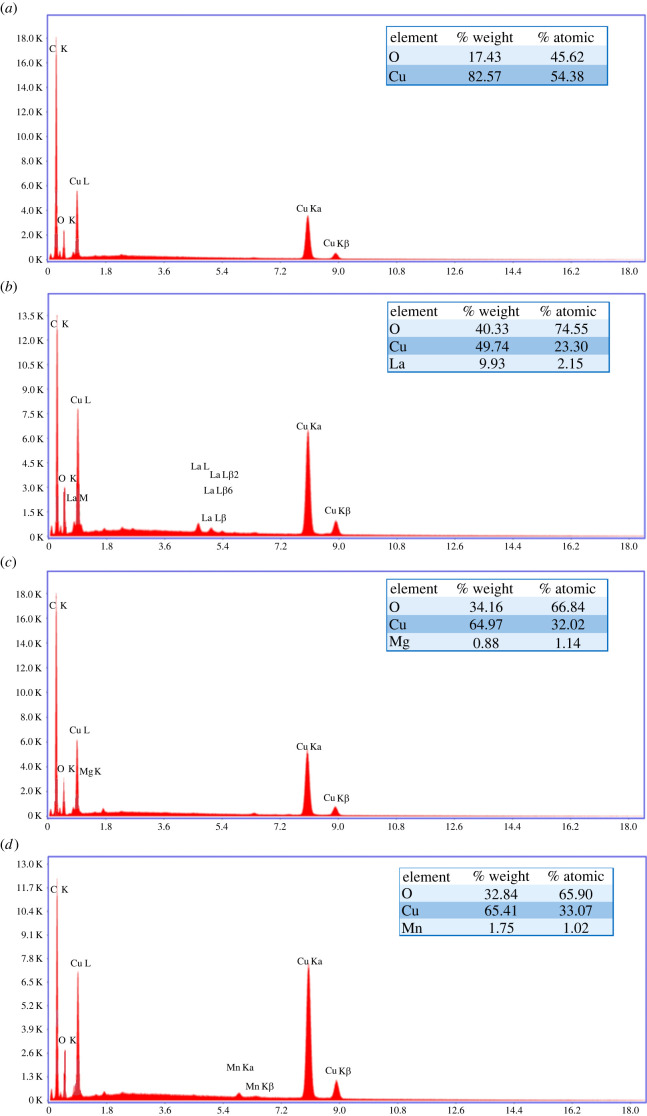
EDS spectra of undoped Cu_2_O Nps (*a*), La-doped Cu_2_O Nps (*b*), Mg-doped Cu_2_O Nps (*c*) and Mn-doped Cu_2_O Nps (*d*).

Elemental analysis of the samples was performed by EDS. The results are shown in figure 1. The EDS spectrum of the undoped Cu_2_O sample shows all K and L emission peaks for copper and oxygen (figure 1*a*). Similar results for Cu_2_O Nps have been previously reported by Mallik *et al.* [57] and Mancier *et al.* [58]. The results obtained by the EDS analysis indicate that the synthesized product is composed of Cu_2_O Nps. Likewise, the calculated Cu : O atomic ratio is approximately 1.2, which is between the values 1.0 and 2.0, theoretical values of CuO and Cu_2_O, respectively. The EDS spectra of the doped Cu_2_O samples show, in addition to the Cu and O peaks, the peaks corresponding to La, Mg and Mn. The average atomic percentages of the doped samples were determined to be 2.2% at. of La (figure 1*b*), 1.1% at. of Mg (figure 1*c*) and 1.0% at. Mn (figure 1*d*). The literature reports a similar atomic % for Mn-doped Cu_2_O Nps obtained by hydrothermal method [37].

**Figure 2.**
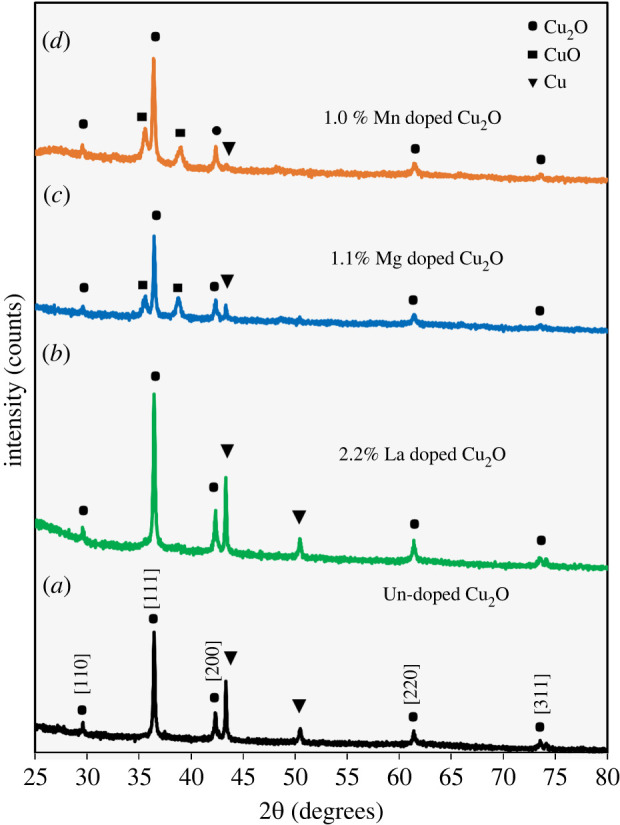
XRD patterns of undoped Cu_2_O Nps (*a*), La-doped Cu_2_O Nps (*b*), Mg-doped Cu_2_O Nps (*c*) and Mn-doped Cu_2_O Nps (*d*).

By means of XRD, it was possible to obtain information on the crystal structure of the synthesized Nps. The recorded XRD spectra are shown in figure 2. For the analysed samples, the spectra reveal diffraction peaks at 2*θ* = 29.6°, 36.5°, 42.3°, 61.4° and 77.4°, which correspond to the structure of cubic cuprite (Cu_2_O) according to JCPDS card no. 005-0667. Therefore, the formation of Cu_2_O with cuprite structure (space group Pn3m, *a* = 4.23 Å) is confirmed. Similar XRD results of undoped [20,21,58–64] and Mn-doped [37,38] Cu_2_O Nps have been previously reported. Likewise, other diffraction peaks were observed at 2*θ* = 43.3° and 50.5° arising from Cu (JCPDS card no. 85-1326) [58].

In addition, in some spectra, peaks at 2*θ* = 35.6° and 38.8° are identified, which would indicate the formation of CuO (JCPDS card no. 45-0937). This is evident in the samples of Cu_2_O doped with Mg (figure 2*c*) and Cu_2_O doped with Mn (figure 2*d*). In addition, no additional diffraction peaks related to impurities of MgO, MnO, MnO_2_, La_2_O_3_, among others, were observed in the doped samples. In this sense, we can affirm that due to the low concentrations of the doping elements used, the La, Mg and Mn ions have been incorporated in the form of ions into the crystal lattice of the Cu_2_O without changing the cubic structure [38].

**Figure 3.**
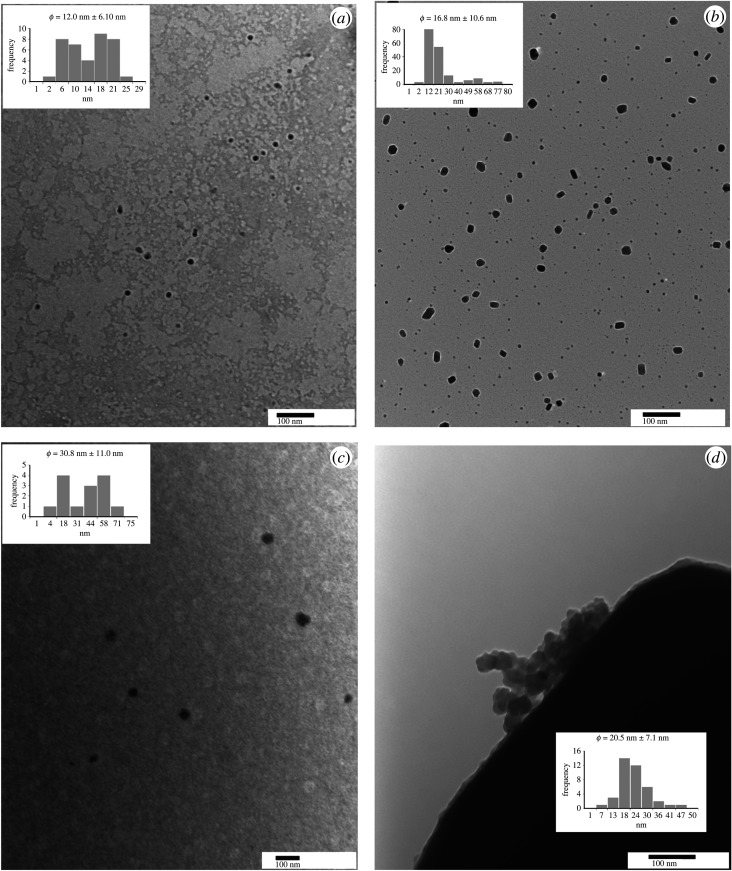
TEM images and particle size distributions of undoped Cu_2_O Nps (*a*), La-doped Cu_2_O Nps (*b*), Mg-doped Cu_2_O Nps (*c*) and Mn-doped Cu_2_O Nps (*d*).

The average size and morphology of undoped and doped Cu_2_O Nps were analysed by TEM. The morphology and particle size distribution of each sample are shown in figure 3. ImageJ software was used to estimate the mean diameter of the Nps. In this case, the diameters of at least 100 particles from each sample were measured. Figure 3*a* shows the size distribution of undoped Cu_2_O Nps. Semi-spherical Nps with diameters from 2.0 nm to 26.0 nm and an average diameter of 12.0 nm ± 6.1 nm can be observed. This result is in agreement with that reported by Yin *et al*. [65], Lai *et al*. [66] and Liu *et al*. [67], who obtained semi-spherical Cu_2_O Nps with similar mean diameters by consecutive oxidation of copper Nps formed in an emulsion. Based on the particle size distribution, we were able to estimate that around 40–45% of Nps could be considered as quantum dots (QDs) (size ≤ 10 nm).

On the other hand, Cu_2_O Nps doped with different elements show a greater variety of shapes. The sample doped with La shows Nps with semi-spherical, hexagonal, truncated cube and elongated shapes (figure 3*b*). These particles have various sizes with a bimodal distribution with mean diameters ranging from 2 nm to 78 nm. The estimated mean diameter was 16.8 nm ± 10.6 nm.

A bimodal distribution is also observed for Mg-doped Cu_2_O Nps (figure 3*c*). This sample has a mean diameter of 30.8 nm ± 11.0 nm. The particles resemble hemispheres that vary in dimensions from 3 nm to 72 nm. The size distribution is illustrated in the histogram at the top left of this figure.

The size distribution of the Mn-doped Cu_2_O Nps is shown in the lower right of figure 3*d*. This figure shows a particle size distribution from 5 to 48 nm with a mean diameter of 20.5 nm ± 7.1 nm. As we can observe, comparing the TEM images, larger semi-spherical Nps are obtained when Mg and Mn are used as doping elements. Likewise, a diversity of forms is obtained when the dopant element is La. The percentages of QDs (size ≤ 10 nm) estimated for the Nps doped with La, Mg and Mn were approximately 37%, approximately 29% and 8%, respectively. A summary of the Np sizes obtained by TEM is presented in table 1.

**Table 1.** Mean diameter and band gap energy size of each sample.

sample	mean diameter (nm)	band gap energy (eV)
Cu_2_O	12.0 ± 6.1	2.433
La-doped Cu_2_O	16.8 ± 10.6	2.425
Mg-doped Cu_2_O	30.8 ± 11.0	2.408
Mn-doped Cu_2_O	20.5 ± 7.1	2.385

The surface area determined using the BET method for the undoped Cu_2_O Nps was determined. The surface area was 11.06 m^2^ g^−1^. This result is better than the values of 0.76 m^2^ g^−1^ and 4.5 m^2^ g^−1^ reported by Bhosale *et al*. [68] and Mrunal *et al*. [69] for Nps of similar sizes, respectively. Unfortunately, due to the small amount of doped Cu_2_O samples, it was not possible to measure their surface area. However, based on the size distribution of the Nps and assuming that they have hemispherical shapes (except for the La-doped sample), the surface area of the doped samples was geometrically estimated. Though this calculation is based on average distribution sizes, it provides a range of estimated surface areas for the doped Nps. The calculated values were 7.63 m^2^ g^−1^ (Cu_2_O:Mn, *ϕ* = 20.5 nm), 7.31 m^2^ g^−1^ (Cu_2_O:La, *ϕ* = 16.8 nm) and 5.09 m^2^ g^−1^ (Cu_2_O:Mg, *ϕ* = 30.8 nm).

**Figure 4.**
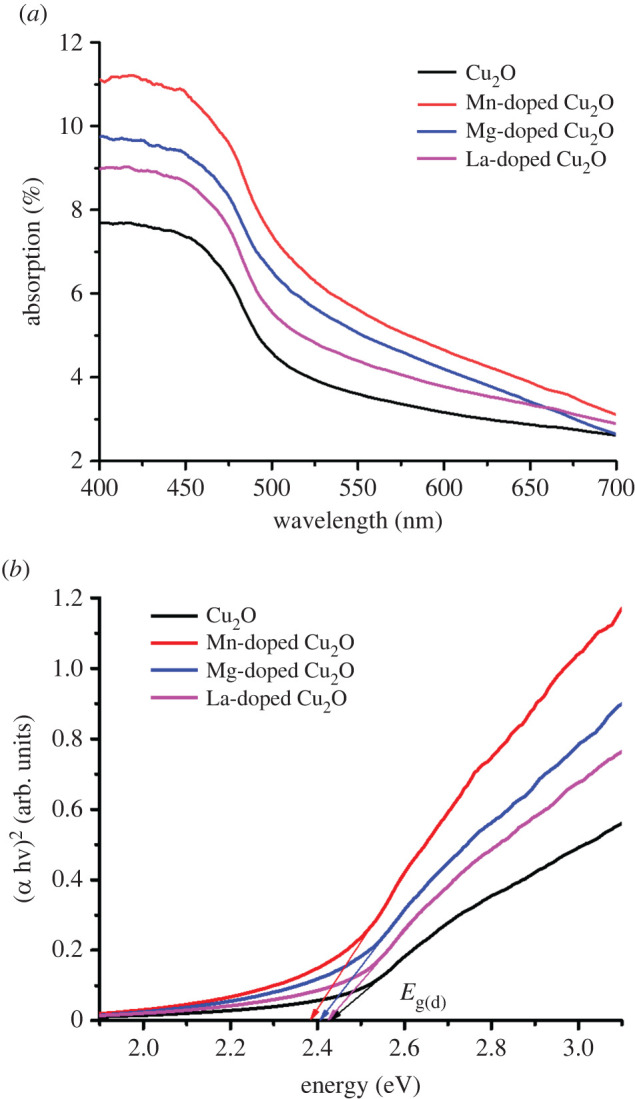
(*a*) UV-VIS spectra and (*b*) direct band gap of undoped and doped cuprous oxide Nps.

Finally, UV-VIS NIR spectroscopic measurements of each sample at room temperature were recorded (figure 4*a*). For semiconductor samples, UV-VIS NIR spectroscopy offers a simple and convenient method to estimate the optical band gap, since this technique probes the electronic transitions between the valence band and the conduction band. The optical direct band gap energy (*E*_g_) of the undoped and La-, Mg- and Mn-doped Cu_2_O Nps was estimated from the UV-VIS absorption spectra. For this, the variation of the absorption coefficient (*α*) with the photon energy was used according to the following relationship:

3.1 $${\left(ah\nu \right)}^{1/p}=A(h\nu -E_{\mathrm{gap}}),$$

where *E*_gap_ is the optical band gap, *A* a constant, *h* the Planck constant, *ν* the frequency of the incident photons and exponent *p* is the transition probability [70,71]. For *p* = 1/2, the transition formalism is direct and allowed.

The values of the optical energy gap were estimated by extrapolation of the linear portion of the graph plotted with (*αhν*)^2^ (figure 4*b*). The optical direct band gap values are summarized in table 1. The direct band gap of undoped Cu_2_O Nps was 2.433 eV, a value higher than the corresponding theoretical band gap of 2.17 eV. However, the experimental value is close to the 2.44 eV reported by Thakur *et al*. [72] for Cu_2_O Nps ranging from 9 nm to 16 nm.

On the other hand, the experimental band gap of Mg-doped Cu_2_O Nps was close to the 2.40 eV reported for Mg-doped Cu_2_O thin layers with larger crystallite size [47]. It is observed that the experimental band gap decreases slightly when a doping element is added. This may be due to the low content of doping element, which modifies only slightly the electronic/optical properties. We must also emphasize that the doped samples show a cubic Cu_2_O structure. Then, in this case, doping produces little structural distortions in the Cu_2_O lattice, and thus *E*_g_ is not severely affected.

Indeed, the dopant element can perform two functions: one is to change the size of the Np, which would mean that the forbidden band would change; the other is when the dopant element is incorporated into the electronic structure of Cu_2_O and thus changes the band gap.

According to the results shown in table 1, we can assume that if the mean diameter increases, the forbidden band decreases, for La-doped Cu_2_O and Mg-doped Cu_2_O. This could be explained by the effects of a quantum confinement due to the small sizes of the crystallites [73]. However, further analysis should be performed as this trend does not apply for Mn-doped Cu_2_O.

## 4. Conclusion

In summary, we report a method that produces doped and undoped Cu_2_O Nps. Doped Nps present a larger average size than undoped Nps. The prepared particles have mean diameters of 12 nm (Cu_2_O), 16.8 nm (La-doped Cu_2_O), 30.8 nm (Mg-doped Cu_2_O) and 20.5 nm (Mn-doped Cu_2_O). It was found that the morphology was changed depending on the identity of doping element. Depending on the dopant element, semi-spherical or elongated Nps similar to nanorods were obtained. In this case, the influence of La on the morphology of Nps is more evident. The energy of the band gap decreases slightly with the doped Nps, being more evident the case of Mn-doped Cu_2_O Nps. On the other hand, the doped cuprous oxide band gap did not show a significant change that made it widen or narrow and remained within the normal range of cuprous oxide optical band gap around 2.433 eV. The optical properties of doped and undoped Cu_2_O Nps confirm the potential of this material for low-cost optoelectronic device applications.

## Data Availability

The datasets supporting this article have been uploaded as part of the electronic supplementary material [74].
